# (*Z*)-1-(2,4-Dimethyl­phen­yl)-3-phenyl-2-(1*H*-1,2,4-triazol-1-yl)prop-2-en-1-one

**DOI:** 10.1107/S1600536812001390

**Published:** 2012-01-21

**Authors:** Rui-Zhu Mu

**Affiliations:** a School of Chemistry and Chemical Engineering, Southwest University, Chongqing 400715, People’s Republic of China

## Abstract

In the title compound, C_19_H_17_N_3_O, the triazole and benzene rings adopt a *Z* configuration with respect to the C=C bond. The phenyl and benzene rings form dihedral angles of 66.20 (9) and 14.36 (9)°, respectively, with the triazole ring. The dihedral angle between the phenyl and benzene rings is 52.64 (8)°.

## Related literature

For the synthesis, see: Wang *et al.* (2009 ▶). For the pharmacological activity of triazole derivatives, see: Zhou & Wang (2012 ▶). For related structures, see: Wang *et al.* (2009 ▶); Yan *et al.* (2009 ▶).
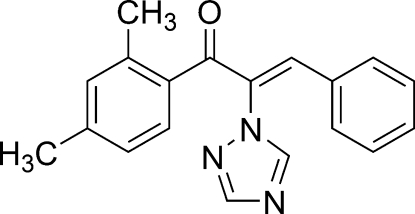


## Experimental

### 

#### Crystal data

C_19_H_17_N_3_O
*M* *_r_* = 303.36
Monoclinic, 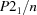

*a* = 12.8499 (3) Å
*b* = 7.8836 (2) Å
*c* = 16.5274 (4) Å
β = 108.376 (1)°
*V* = 1588.91 (7) Å^3^
*Z* = 4
Mo *K*α radiation
μ = 0.08 mm^−1^
*T* = 296 K
0.20 × 0.20 × 0.18 mm

#### Data collection

Bruker SMART CCD diffractometer
Absorption correction: multi-scan (*SADABS*; Sheldrick, 1996 ▶) *T*_min_ = 0.984, *T*_max_ = 0.986
11768 measured reflections
2796 independent reflections
2369 reflections with *I* > 2σ(*I*)
*R*_int_ = 0.025

#### Refinement

*R*[*F* ^2^ > 2σ(*F* ^2^)] = 0.041
*wR*(*F* ^2^) = 0.113
*S* = 1.04
2796 reflections
215 parameters
H atoms treated by a mixture of independent and constrained refinement
Δρ_max_ = 0.18 e Å^−3^
Δρ_min_ = −0.15 e Å^−3^

### 

Data collection: *SMART* (Bruker, 2001 ▶); cell refinement: *SAINT* (Bruker, 2001 ▶); data reduction: *SAINT*; program(s) used to solve structure: *SHELXS97* (Sheldrick, 2008 ▶); program(s) used to refine structure: *SHELXL97* (Sheldrick, 2008 ▶); molecular graphics: *PLATON* (Spek, 2009 ▶); software used to prepare material for publication: *SHELXTL* (Sheldrick, 2008 ▶).

## Supplementary Material

Crystal structure: contains datablock(s) global, I. DOI: 10.1107/S1600536812001390/lh5401sup1.cif

Structure factors: contains datablock(s) I. DOI: 10.1107/S1600536812001390/lh5401Isup2.hkl

Supplementary material file. DOI: 10.1107/S1600536812001390/lh5401Isup3.cml

Additional supplementary materials:  crystallographic information; 3D view; checkCIF report

## Figures and Tables

**Fig. 1.**
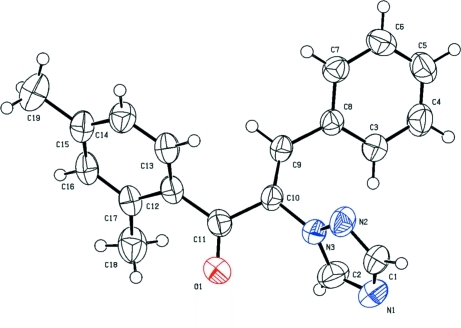
The molecular structure of (I), showing displacement ellipsoids drawn at the 50% probability level.

## supplementary crystallographic information

### Comment

Chalcones are an important type of biologically active compounds with a diaryl enone structural unit. Triazole compounds exhibit a broad bioactive spectrum (Zhou & Wang, 2012). Our interest is to develop novel triazole-derived chalcones as medicinal agents. Examples of related structures of triazolylchalcones have been reported (Wang *et al.*, 2009; Yan *et al.*, 2009). Herein, the crystal structure of the title compound (I) is reported.

In the molecular structure of (I) (Fig. 1) the triazole and benzene rings adopt a *Z* configuration with respect to the C═C bond. The phenyl (C3-C8) and benzene (C12-C17) rings form dihedral angles of 66.20 (9) and 14.36 (9)°, respectively with the triazole ring (N1-N3/C1/C2). The dihedral angle between the phenyl and benzene rings is 52.64 (8)Å.

### Experimental

The title compound was prepared according to the procedure of Wang *et al.* (2009). A mixture of 1-(2,4-dimethylphenyl)-2-(1H-1,2,4-triazol-1-yl) ethanone (1.08 g, 5.0 mmol) and benzaldehyde (0.74 g, 7.0 mmol) in toluene (30 mL) in the presence of glacial acetic acid (0.08 mL, 1.4 mmol) and piperidine (0.08 mL, 1.4 mmol) as catalyst was refluxed. After the reaction was completed (monitored by TLC, petroleum ether/ethyl acetate, 3/1, V/V), the solvent was removed. The residue was dissolved in dichloromethane (30 mL) and washed with water (3x30 mL). The resulting phase was dried over anhydrous sodium sulfate, concentrated under reduced pressure and then purified by silica gel column chromatography eluting with petroleum ether/ethyl acetate (10/1-2/1, V/V) to give the title compound (I) (0.923 g) as solid. Mp.396-397K. A crystal of (I) suitable for X-ray analysis was grown from a mixed solution of ethyl acetate and petroleum ether by slow evaporation at room temperature.

### Refinement

H atoms were placed in calculated positions with C—H = 0.93Å (aromatic) and 0.96Å (methyl). The *U*_iso_(H) values were set equal to 1.2U_eq_(C_aromatic_) and 1.5U_eq_(C_methyl_). The H atom bonded to C9 was refined independently with an isotropic displacement paramemeter.

### Crystal data

**Table:**

C_19_H_17_N_3_O	*F*(000) = 640
*M**_r_* = 303.36	*D*_x_ = 1.268 Mg m^−^^3^
Monoclinic, *P*2_1_/*n*	Mo *K*α radiation, λ = 0.71073 Å
Hall symbol: -P 2yn	Cell parameters from 4934 reflections
*a* = 12.8499 (3) Å	θ = 2.4–27.1°
*b* = 7.8836 (2) Å	µ = 0.08 mm^−^^1^
*c* = 16.5274 (4) Å	*T* = 296 K
β = 108.376 (1)°	Block, colorless
*V* = 1588.91 (7) Å^3^	0.20 × 0.20 × 0.18 mm
*Z* = 4	

### Data collection

**Table:**

Bruker SMART CCD diffractometer	2796 independent reflections
Radiation source: fine-focus sealed tube	2369 reflections with *I* > 2σ(*I*)
graphite	*R*_int_ = 0.025
φ and ω scans	θ_max_ = 25.0°, θ_min_ = 1.8°
Absorption correction: multi-scan (*SADABS*; Sheldrick, 1996)	*h* = −15→9
*T*_min_ = 0.984, *T*_max_ = 0.986	*k* = −8→9
11768 measured reflections	*l* = −17→19

### Refinement

**Table:**

Refinement on *F*^2^	Secondary atom site location: difference Fourier map
Least-squares matrix: full	Hydrogen site location: inferred from neighbouring sites
*R*[*F*^2^ > 2σ(*F*^2^)] = 0.041	H atoms treated by a mixture of independent and constrained refinement
*wR*(*F*^2^) = 0.113	*w* = 1/[σ^2^(*F*_o_^2^) + (0.0517*P*)^2^ + 0.3715*P*] where *P* = (*F*_o_^2^ + 2*F*_c_^2^)/3
*S* = 1.04	(Δ/σ)_max_ = 0.004
2796 reflections	Δρ_max_ = 0.18 e Å^−^^3^
215 parameters	Δρ_min_ = −0.15 e Å^−^^3^
0 restraints	Extinction correction: *SHELXL97* (Sheldrick, 2008), Fc^*^=kFc[1+0.001xFc^2^λ^3^/sin(2θ)]^-1/4^
Primary atom site location: structure-invariant direct methods	Extinction coefficient: 0.017 (2)

### Special details

**Table:**

Geometry. All e.s.d.'s (except the e.s.d. in the dihedral angle between two l.s. planes) are estimated using the full covariance matrix. The cell e.s.d.'s are taken into account individually in the estimation of e.s.d.'s in distances, angles and torsion angles; correlations between e.s.d.'s in cell parameters are only used when they are defined by crystal symmetry. An approximate (isotropic) treatment of cell e.s.d.'s is used for estimating e.s.d.'s involving l.s. planes.
Refinement. Refinement of *F*^2^ against ALL reflections. The weighted *R*-factor *wR* and goodness of fit *S* are based on *F*^2^, conventional *R*-factors *R* are based on *F*, with *F* set to zero for negative *F*^2^. The threshold expression of *F*^2^ > σ(*F*^2^) is used only for calculating *R*-factors(gt) *etc*. and is not relevant to the choice of reflections for refinement. *R*-factors based on *F*^2^ are statistically about twice as large as those based on *F*, and *R*- factors based on ALL data will be even larger.

### Fractional atomic coordinates and isotropic or equivalent isotropic displacement parameters (Å^2^)

**Table:**

	*x*	*y*	*z*	*U*_iso_*/*U*_eq_	
C1	0.20259 (13)	1.1290 (2)	0.85885 (11)	0.0604 (5)	
H1	0.1666	1.2043	0.8842	0.072*	
C2	0.23209 (14)	0.9674 (3)	0.77106 (11)	0.0636 (5)	
H2	0.2254	0.9031	0.7225	0.076*	
C3	0.29220 (13)	0.6718 (2)	0.94192 (10)	0.0531 (4)	
H3	0.2511	0.7004	0.8865	0.064*	
C4	0.24256 (14)	0.5912 (2)	0.99443 (12)	0.0634 (5)	
H4	0.1685	0.5637	0.9736	0.076*	
C5	0.30203 (15)	0.5514 (2)	1.07733 (11)	0.0615 (5)	
H5	0.2679	0.4990	1.1126	0.074*	
C6	0.41158 (15)	0.5892 (2)	1.10754 (10)	0.0578 (4)	
H6	0.4518	0.5634	1.1636	0.069*	
C7	0.46229 (13)	0.6653 (2)	1.05485 (9)	0.0479 (4)	
H7	0.5372	0.6870	1.0753	0.057*	
C8	0.40332 (11)	0.71002 (18)	0.97182 (9)	0.0413 (3)	
C9	0.46292 (12)	0.78548 (19)	0.91780 (9)	0.0428 (4)	
C10	0.42801 (11)	0.89791 (19)	0.85496 (9)	0.0417 (3)	
C11	0.48890 (13)	0.9480 (2)	0.79522 (10)	0.0486 (4)	
C12	0.60794 (12)	0.90705 (19)	0.81731 (9)	0.0450 (4)	
C13	0.67846 (13)	0.9510 (2)	0.89704 (10)	0.0511 (4)	
H13	0.6502	1.0017	0.9365	0.061*	
C14	0.78937 (13)	0.9210 (2)	0.91891 (11)	0.0566 (4)	
H14	0.8351	0.9543	0.9723	0.068*	
C15	0.83373 (13)	0.8419 (2)	0.86257 (11)	0.0543 (4)	
C16	0.76298 (13)	0.7989 (2)	0.78301 (10)	0.0531 (4)	
H16	0.7918	0.7458	0.7446	0.064*	
C17	0.65123 (12)	0.8313 (2)	0.75783 (10)	0.0481 (4)	
C18	0.58064 (16)	0.7803 (3)	0.67006 (11)	0.0724 (6)	
H18A	0.6255	0.7291	0.6400	0.109*	
H18B	0.5449	0.8788	0.6396	0.109*	
H18C	0.5264	0.7003	0.6746	0.109*	
C19	0.95482 (15)	0.8037 (3)	0.88741 (15)	0.0825 (6)	
H19A	0.9910	0.8916	0.8663	0.124*	
H19B	0.9656	0.6968	0.8633	0.124*	
H19C	0.9849	0.7983	0.9484	0.124*	
H1M	0.5369 (13)	0.746 (2)	0.9311 (9)	0.045 (4)*	
N1	0.15299 (12)	1.0603 (2)	0.78249 (9)	0.0674 (4)	
N2	0.30462 (10)	1.08424 (18)	0.89592 (8)	0.0542 (4)	
N3	0.32284 (9)	0.97702 (16)	0.83766 (7)	0.0434 (3)	
O1	0.43871 (10)	1.0182 (2)	0.72867 (8)	0.0757 (4)	

### Atomic displacement parameters (Å^2^)

**Table:**

	*U*^11^	*U*^22^	*U*^33^	*U*^12^	*U*^13^	*U*^23^
C1	0.0483 (9)	0.0687 (12)	0.0681 (10)	0.0169 (8)	0.0240 (8)	0.0059 (9)
C2	0.0531 (10)	0.0799 (13)	0.0503 (9)	0.0104 (9)	0.0054 (7)	−0.0035 (8)
C3	0.0446 (9)	0.0601 (10)	0.0544 (9)	−0.0009 (8)	0.0151 (7)	0.0054 (8)
C4	0.0486 (10)	0.0656 (12)	0.0804 (12)	−0.0063 (8)	0.0267 (9)	0.0067 (9)
C5	0.0705 (12)	0.0555 (10)	0.0703 (11)	0.0024 (9)	0.0390 (9)	0.0125 (8)
C6	0.0705 (12)	0.0536 (10)	0.0511 (9)	0.0068 (8)	0.0219 (8)	0.0097 (7)
C7	0.0460 (9)	0.0442 (9)	0.0527 (8)	0.0036 (7)	0.0144 (7)	0.0023 (7)
C8	0.0418 (8)	0.0375 (8)	0.0479 (8)	0.0051 (6)	0.0188 (6)	0.0002 (6)
C9	0.0380 (8)	0.0447 (8)	0.0482 (8)	0.0046 (7)	0.0174 (6)	−0.0012 (6)
C10	0.0368 (7)	0.0458 (8)	0.0449 (7)	0.0032 (6)	0.0166 (6)	−0.0011 (6)
C11	0.0487 (9)	0.0509 (9)	0.0509 (8)	0.0026 (7)	0.0225 (7)	0.0035 (7)
C12	0.0467 (8)	0.0428 (8)	0.0525 (8)	−0.0021 (7)	0.0259 (7)	0.0016 (7)
C13	0.0521 (9)	0.0533 (10)	0.0552 (9)	−0.0002 (7)	0.0272 (7)	−0.0055 (7)
C14	0.0488 (9)	0.0617 (11)	0.0599 (9)	−0.0061 (8)	0.0182 (7)	−0.0072 (8)
C15	0.0474 (9)	0.0499 (10)	0.0720 (10)	−0.0020 (7)	0.0280 (8)	−0.0005 (8)
C16	0.0565 (10)	0.0478 (9)	0.0672 (10)	−0.0023 (8)	0.0372 (8)	−0.0056 (8)
C17	0.0507 (9)	0.0455 (9)	0.0561 (9)	−0.0066 (7)	0.0284 (7)	−0.0030 (7)
C18	0.0682 (12)	0.0892 (15)	0.0663 (11)	−0.0097 (10)	0.0308 (9)	−0.0223 (10)
C19	0.0525 (11)	0.0907 (16)	0.1073 (16)	0.0077 (11)	0.0292 (11)	−0.0055 (13)
N1	0.0491 (8)	0.0839 (11)	0.0649 (9)	0.0173 (8)	0.0117 (7)	0.0101 (8)
N2	0.0462 (8)	0.0604 (9)	0.0585 (8)	0.0086 (6)	0.0201 (6)	−0.0077 (6)
N3	0.0397 (7)	0.0489 (7)	0.0427 (6)	0.0051 (5)	0.0145 (5)	0.0015 (5)
O1	0.0634 (8)	0.1056 (11)	0.0651 (8)	0.0179 (7)	0.0302 (6)	0.0344 (7)

### Geometric parameters (Å, °)

**Table:**

C1—N2	1.308 (2)	C10—C11	1.4933 (19)
C1—N1	1.336 (2)	C11—O1	1.2186 (18)
C1—H1	0.9300	C11—C12	1.492 (2)
C2—N1	1.314 (2)	C12—C13	1.387 (2)
C2—N3	1.330 (2)	C12—C17	1.406 (2)
C2—H2	0.9300	C13—C14	1.376 (2)
C3—C4	1.383 (2)	C13—H13	0.9300
C3—C8	1.389 (2)	C14—C15	1.384 (2)
C3—H3	0.9300	C14—H14	0.9300
C4—C5	1.378 (2)	C15—C16	1.384 (2)
C4—H4	0.9300	C15—C19	1.509 (2)
C5—C6	1.370 (2)	C16—C17	1.387 (2)
C5—H5	0.9300	C16—H16	0.9300
C6—C7	1.379 (2)	C17—C18	1.503 (2)
C6—H6	0.9300	C18—H18A	0.9600
C7—C8	1.388 (2)	C18—H18B	0.9600
C7—H7	0.9300	C18—H18C	0.9600
C8—C9	1.4727 (19)	C19—H19A	0.9600
C9—C10	1.331 (2)	C19—H19B	0.9600
C9—H1M	0.958 (15)	C19—H19C	0.9600
C10—N3	1.4330 (18)	N2—N3	1.3560 (17)
			
N2—C1—N1	116.25 (15)	C13—C12—C11	119.40 (13)
N2—C1—H1	121.9	C17—C12—C11	121.46 (14)
N1—C1—H1	121.9	C14—C13—C12	121.27 (14)
N1—C2—N3	111.45 (16)	C14—C13—H13	119.4
N1—C2—H2	124.3	C12—C13—H13	119.4
N3—C2—H2	124.3	C13—C14—C15	120.85 (15)
C4—C3—C8	120.15 (15)	C13—C14—H14	119.6
C4—C3—H3	119.9	C15—C14—H14	119.6
C8—C3—H3	119.9	C14—C15—C16	117.57 (15)
C5—C4—C3	120.50 (16)	C14—C15—C19	120.99 (16)
C5—C4—H4	119.7	C16—C15—C19	121.44 (16)
C3—C4—H4	119.7	C15—C16—C17	123.21 (14)
C6—C5—C4	119.79 (15)	C15—C16—H16	118.4
C6—C5—H5	120.1	C17—C16—H16	118.4
C4—C5—H5	120.1	C16—C17—C12	117.96 (14)
C5—C6—C7	120.03 (15)	C16—C17—C18	119.53 (14)
C5—C6—H6	120.0	C12—C17—C18	122.48 (14)
C7—C6—H6	120.0	C17—C18—H18A	109.5
C6—C7—C8	121.04 (15)	C17—C18—H18B	109.5
C6—C7—H7	119.5	H18A—C18—H18B	109.5
C8—C7—H7	119.5	C17—C18—H18C	109.5
C7—C8—C3	118.45 (13)	H18A—C18—H18C	109.5
C7—C8—C9	118.42 (13)	H18B—C18—H18C	109.5
C3—C8—C9	123.01 (13)	C15—C19—H19A	109.5
C10—C9—C8	129.07 (14)	C15—C19—H19B	109.5
C10—C9—H1M	117.6 (9)	H19A—C19—H19B	109.5
C8—C9—H1M	113.3 (9)	C15—C19—H19C	109.5
C9—C10—N3	120.68 (13)	H19A—C19—H19C	109.5
C9—C10—C11	125.02 (13)	H19B—C19—H19C	109.5
N3—C10—C11	114.25 (12)	C2—N1—C1	101.62 (14)
O1—C11—C12	121.95 (13)	C1—N2—N3	101.96 (13)
O1—C11—C10	118.58 (14)	C2—N3—N2	108.70 (13)
C12—C11—C10	119.45 (13)	C2—N3—C10	131.78 (13)
C13—C12—C17	119.09 (14)	N2—N3—C10	119.52 (11)
			
C8—C3—C4—C5	1.4 (3)	C12—C13—C14—C15	−1.6 (3)
C3—C4—C5—C6	−1.1 (3)	C13—C14—C15—C16	1.8 (3)
C4—C5—C6—C7	−0.6 (3)	C13—C14—C15—C19	−178.00 (17)
C5—C6—C7—C8	2.2 (3)	C14—C15—C16—C17	0.0 (3)
C6—C7—C8—C3	−1.9 (2)	C19—C15—C16—C17	179.80 (16)
C6—C7—C8—C9	−177.89 (14)	C15—C16—C17—C12	−1.9 (2)
C4—C3—C8—C7	0.1 (2)	C15—C16—C17—C18	179.96 (16)
C4—C3—C8—C9	175.91 (15)	C13—C12—C17—C16	2.1 (2)
C7—C8—C9—C10	−149.02 (16)	C11—C12—C17—C16	179.33 (14)
C3—C8—C9—C10	35.2 (2)	C13—C12—C17—C18	−179.86 (16)
C8—C9—C10—N3	7.0 (2)	C11—C12—C17—C18	−2.6 (2)
C8—C9—C10—C11	−170.43 (14)	N3—C2—N1—C1	1.4 (2)
C9—C10—C11—O1	162.17 (16)	N2—C1—N1—C2	−1.1 (2)
N3—C10—C11—O1	−15.4 (2)	N1—C1—N2—N3	0.3 (2)
C9—C10—C11—C12	−16.3 (2)	N1—C2—N3—N2	−1.3 (2)
N3—C10—C11—C12	166.14 (13)	N1—C2—N3—C10	179.01 (15)
O1—C11—C12—C13	131.04 (18)	C1—N2—N3—C2	0.56 (18)
C10—C11—C12—C13	−50.6 (2)	C1—N2—N3—C10	−179.71 (13)
O1—C11—C12—C17	−46.2 (2)	C9—C10—N3—C2	−113.9 (2)
C10—C11—C12—C17	132.18 (16)	C11—C10—N3—C2	63.8 (2)
C17—C12—C13—C14	−0.4 (2)	C9—C10—N3—N2	66.43 (19)
C11—C12—C13—C14	−177.68 (15)	C11—C10—N3—N2	−115.87 (15)
